# Editorial: Aminobiogenic potential of fermented food microbiota

**DOI:** 10.3389/fmicb.2023.1176149

**Published:** 2023-03-23

**Authors:** Małgorzata Karwowska, Joanna Stadnik, Milena Brasca

**Affiliations:** ^1^Department of Meat Technology and Food Quality, University of Life Sciences in Lublin, Lublin, Poland; ^2^Institute of Sciences of Food Production (ISPA), National Research Council (CNR), Milan, Italy

**Keywords:** food microbiota, decarboxylation, biogenic amines, fermented food, lactic acid bacteria

Biogenic amines (BAs) are non-volatile low-molecular-weight nitrogenous organic bases that can be found in nearly all types of foods in a wide and variable range of concentrations. The most significant BAs occurring in food are histamine, tyramine, putrescine, cadaverine, tryptamine, 2-phenylethylamine, spermine, spermidine, and agmatine. BAs are produced by microorganisms, mainly bacteria, through the action of decarboxylases, which act selectively on precursor amino acids in which they remove the carboxyl group with the formation of the correspondent biogenic amine and CO_2_. The decarboxylative pathways are activated for two main physiological reasons. Decarboxylation is one of the responses of cells to acid stress and it has been demonstrated that the transcription of many decarboxylase genes is induced by low pH and improves cell performances in acid conditions. Moreover, these pathways can bring supplementary energy to the cells by energizing the proton motive force associated with the membrane.

BAs can be produced both by Gram-positive and Gram-negative bacteria which occur naturally in food products, are introduced by contamination, or are added as a starter culture. Among Gram-negative bacteria, spoilage microorganisms belonging to Enterobacteriaceae and Pseudomonas are known as the major producers of cadaverine, histamine, and putrescine. The ability to produce BAs is widespread also among Gram-positive bacteria. The decarboxylase activity has been found in strains belonging to the genera Bacillus and Staphylococcus. However, attention has been mainly focused on lactic acid bacteria (LAB), which are commonly present or intentionally used in fermented food.

Ingestion of food containing BAs represents a considerable toxicological risk for consumer health since these compounds can cause headaches, heart palpitations, vomiting, diarrhea, and hypertensive crisis. However, their toxic effect depends on the type of BAs, individual sensitivity or health status, and on the consumption of ethanol or monoamine oxidase inhibitory drugs, which interact with amino oxidase enzymatic systems responsible for the detoxification process of exogenous BAs.

Microorganisms (used as starter cultures, as well as those naturally occurring during ripening or in spontaneous fermentations) that are able to produce biogenic amines in models or real systems may be evaluated. Furthermore, such subjects could include descriptions of novel knowledge and strategies for aminogenesis control: use of bioprotective cultures producing bacteriocins or other antimicrobial substances, technological additives, effects of packaging, other non-thermal treatments, and use of microbial cultures able to degrade BAs and detoxifying them through the action of amino oxidases.

In this context, we organized this Research Topic on “*The Aminobiogenic Potential of Fermented Food Microbiota*”. The topic collected four research articles from international researchers. This Research Topic is focused on the aminobiogenic potential of fermented food microbiota, taking into consideration the conditions affecting biogenic amine accumulation and the enzymes and genes involved in the biosynthetic mechanisms.

The study of Sang et al. aimed to evaluate the ability to produce BAs of halophilic bacteria isolated from grasshopper sub shrimp paste. The authors analyzed and discussed the bacterial and fungal diversities and BAs contents in grasshopper sub shrimp paste at different fermentation times. They concluded that the actual production of BA by a particular strain was closely related to other species present in the complex fermentation system. The experiment carried out provided important insights into the microbiota and BAs content of grasshopper sub shrimp paste.

Yang et al. evaluated the amine production abilities of strains of enteric bacteria screened from Chinese traditional fermented fish. The genotypic diversity of amino acid decarboxylase and the effect of pH were explored on strains of high-yield BAs. They reported that all tested strains carried the corresponding amino acid decarboxylase genes of putrescine and cadaverine. In addition, almost half of the strains carried the histidine decarboxylase gene. The research demonstrates that acid stress causes growth delay but can increase the contents of putrescine, histamine, and cadaverine.

Yu et al. presented a study on the change in BA content and microbes involved in BA formation during mustard fermentation. They showed that diverse bacterial species harbored genes associated with the production of various BAs. Four BAs (cadaverine, putrescine, tyramine, and histamine) were generated during mustard fermentation. According to the metagenome sequencing, the predominant genus was *Bacillus* followed by *Lactobacillus, Weissella*, and *Leuconostoc* in the initial fermentation stage, while *Lactobacillus* became the most dominant genus in the late stage.

Hernández-Macias et al. investigated whether the use of cava lees can help to control biogenic amine formation in bread and fermented sausages. The authors showed that cava lees and their phenolic extract are an effective strategy to control the undesirable accumulation of high levels of biogenic amines during the production of fermented products. However, the effect of reducing the high levels of biogenic amines was not as effective as the use of starter cultures.

## Author contributions

All authors listed have made a substantial, direct, and intellectual contribution to the work and approved it for publication.

